# Supplementary material for: Comparison of COVID-19 outcomes among shielded and non-shielded populations
Source: Sci Rep. 2021 Jul 27;11:15278. doi: 10.1038/s41598-021-94630-6 (PMC8316565; doi:10.1038/s41598-021-94630-6)
Supplement: Supplementary file 1 — Supplementary Information. [file 41598_2021_94630_MOESM1_ESM.docx]

**Comparison of COVID-19 outcomes among shielded and non-shielded populations:**

**A general population cohort study of 1.3 million**

Bhautesh D Jani^†^, Frederick K Ho^†^, David J Lowe, Jamie P Traynor, Sean MacBride-Stewart, Patrick B Mark, Frances S Mair, Jill P Pell*

^†^Joint-first author

Bhautesh D Jani PhD

Clinical Senior Lecturer in General Practice and Primary Care

Institute of Health and Wellbeing, University of Glasgow

Glasgow G12 9LX, UK

[Bhautesh.jani@glasgow.ac.uk](mailto:Bhautesh.jani@glasgow.ac.uk)

Frederick K Ho PhD

Research Associate

Institute of Health and Wellbeing, University of Glasgow

Glasgow, G12 8RZ, UK

[Frederick.Ho@glasgow.ac.uk](mailto:Frederick.Ho@glasgow.ac.uk)

David J Lowe MSc

Consultant in Emergency Medicine

Queen Elizabeth University Hospital, NHS Greater Glasgow and Clyde

Glasgow, G52 4TF, UK

[David.lowe@nhs.net](mailto:David.lowe@nhs.net)

Jamie P Traynor MD

Consultant Nephrologist

Queen Elizabeth University Hospital, NHS Greater Glasgow and Clyde

Glasgow, G52 4TF, UK

[Jamie.traynor@ggc.scot.nhs.uk](mailto:Jamie.traynor@ggc.scot.nhs.uk)

Sean MacBride-Stewart PhD

Lead Pharmacist (Medicines Management Resources)

Pharmacy Services, NHS Greater Glasgow and Clyde

Glasgow, G76 7AT, UK

[Sean.MacBride-Stewart@ggc.scot.nhs.uk](mailto:Sean.MacBride-Stewart@ggc.scot.nhs.uk)

Patrick B Mark PhD

Professor of Nephrology

Institute of Cardiovascular and Medical Sciences, University of Glasgow

Glasgow, G12 8TA, UK

[Patrick.Mark@glasgow.ac.uk](mailto:Patrick.Mark@glasgow.ac.uk)

Frances S Mair MD

Norie Miller Professor of General Practice

Institute of Health and Wellbeing, University of Glasgow

Glasgow G12 9LX, UK

[Frances.mair@glasgow.ac.uk](mailto:Frances.mair@glasgow.ac.uk)

Jill P Pell MD

Henry Mechan Professor of Public Health

Institute of Health and Wellbeing, University of Glasgow

Glasgow, G12 8RZ, UK

[Jill.pell@glasgow.ac.uk](mailto:Jill.pell@glasgow.ac.uk)

***Address for correspondence:**

Professor Jill Pell

Director of the Institute of Health and Wellbeing

University of Glasgow

1 Lilybank Gardens

Glasgow G12 8RZ

United Kingdom

[Jill.pell@glasgow.ac.uk](mailto:Jill.pell@glasgow.ac.uk)

**Supplementary Table 1.** Vulnerable Patient List in UK

| **Shielded** | **Moderate Risk** |
| --- | --- |
| Severe respiratory disease:   - severe COPD - severe asthma (high-dose steroids) - cystic fibrosis | Chronic respiratory disease:   - COPD - asthma - emphysema - bronchitis |
| Specific cancers:   - Lung cancer plus radical radiotherapy or active chemotherapy - Blood or bone marrow cancers - Bone marrow or stem cell transplant   - in last 6 months, or   - still taking immunosuppressants - immunotherapy or continuing antibody treatment - protein kinase inhibitors or PARP inhibitors | Other chronic conditions   - heart disease/hypertension - diabetes - kidney disease - liver disease - neurological conditions |
| Pregnant with significant heart disease | Pregnant |
| Immunosuppressive therapy | Weakened immune system due to:   - Medical condition - Medication (e.g. oral steroids, chemotherapy) |
| Solid organ transplant | BMI ≥40 kg/m^2^ |
| Relevant rare diseases and inborn errors of metabolism (e.g. SCID, homozygous sickle cell) | ≥70 years of age |
| Renal dialysis |  |

**Supplementary Table 2.** Associations* between risk categories and risk criteria and population-level COVID-19 outcomes

|  | **Confirmed COVID-19 infection** | | **COVID-19 hospitalisation** | | **COVID-19 ICU admission** | | **COVID-19 mortality** | |
| --- | --- | --- | --- | --- | --- | --- | --- | --- |
|  | **RR (95% CI)** | **P-value** | **RR (95% CI)** | **P-value** | **RR (95% CI)** | **P-value** | **RR (95% CI)** | **P-value** |
| ***Low*** | 1 (Reference) |  | 1 (Reference) |  | 1 (Reference) |  | 1 (Reference) |  |
| ***Moderate*** |  |  |  |  |  |  |  |  |
| *Overall* | 4.11 (3.83-4.42) | <0.0001 | 6.83 (6.09-7.67) | <0.0001 | 2.15 (1.49-3.10) | <0.0001 | 25.41 (20.36-31.71) | <0.0001 |
| Chronic respiratory disease | 2.30 (2.07-2.56) | <0.0001 | 3.76 (3.21-4.41) | <0.0001 | 1.15 (0.62-2.14) | 0.65 | 3.88 (2.77-5.44) | <0.0001 |
| Heart disease | 6.53 (5.83-7.31) | <0.0001 | 11.63 (9.93-13.63) | <0.0001 | 1.92 (0.88-4.20) | 0.1 | 26.00 (19.71-34.31) | <0.0001 |
| Hypertension | 4.60 (4.22-5.01) | <0.0001 | 8.01 (7.04-9.11) | <0.0001 | 2.44 (1.55-3.85) | 0.0001 | 15.68 (12.24-20.09) | <0.0001 |
| Diabetes | 5.59 (5.02-6.23) | <0.0001 | 10.49 (9.03-12.19) | <0.0001 | 4.89 (3.03-7.89) | <0.0001 | 16.23 (12.16-21.66) | <0.0001 |
| Weakened immune system | 7.79 (4.43-13.72) | <0.0001 | 19.62 (10.50-36.69) | <0.0001 | - | - | 56.19 (24.85-127.02) | <0.0001 |
| ≥70 years of age | 8.08 (7.48-8.72) | <0.0001 | 14.96 (13.29-16.85) | <0.0001 | 0.90 (0.43-1.86) | 0.77 | 72.98 (58.81-90.55) | <0.0001 |
| ***Shielded*** |  |  |  |  |  |  |  |  |
| *Overall* | 8.45 (7.44-9.59) | <0.0001 | 19.35 (16.45-22.77) | <0.0001 | 2.78 (1.12-6.91) | 0.03 | 57.56 (44.06-75.19) | <0.0001 |
| Severe respiratory disease | 6.79 (5.78-7.99) | <0.0001 | 16.40 (13.48-19.95) | <0.0001 | 0.82 (0.11-5.89) | 0.84 | 52.85 (39.31-71.05) | <0.0001 |
| Specific cancers | 12.13 (8.83-16.65) | <0.0001 | 27.21 (18.69-39.63) | <0.0001 | - | - | 71.72 (42.36-121.42) | <0.0001 |
| Immunosuppressive therapy | 9.25 (7.21-11.88) | <0.0001 | 21.85 (16.30-29.29) | <0.0001 | 8.40 (2.64-26.72) | 0.0003 | 52.23 (33.64-81.08) | <0.0001 |
| Solid organ transplant | 10.90 (6.77-17.55) | <0.0001 | 27.77 (16.61-46.44) | <0.0001 | 10.84 (1.50-78.16) | 0.02 | 62.03 (29.03-132.56) | <0.0001 |
| Rare diseases and IEM | 15.91 (11.44-22.12) | <0.0001 | 31.45 (20.86-47.42) | <0.0001 | 8.18 (1.13-58.93) | 0.04 | 132.29 (82.60-211.86) | <0.0001 |
| Renal dialysis | 50.29 (35.06-72.12) | <0.0001 | 84.41 (52.70-135.20) | <0.0001 | - | - | 158.41 (74.11-338.62) | <0.0001 |

*adjusted for sex, deprivation quintile, and other risk categories

RR relative risk; CI confidence interval; IEM inborn errors of metabolism

**Supplementary Table 3.** Associations* between risk categories and risk criteria and COVID-19 outcomes among confirmed cases

|  | **COVID-19 hospitalisation** | | **COVID-19 ICU admission** | | **COVID-19 case-fatality** | |
| --- | --- | --- | --- | --- | --- | --- |
|  | **N=3,348^†^** | | **N=3,348^†^** | | **N=3,846^‡^** | |
|  | **RR (95% CI)** | **P-value** | **RR (95% CI)** | **P-value** | **RR (95% CI)** | **P-value** |
| ***Low*** | 1 (Reference) |  | 1 (Reference) |  | 1 (Reference) |  |
| ***Moderate*** |  |  |  |  |  |  |
| *Overall* | 1.34 (1.23-1.46) | <0.0001 | 0.40 (0.28-0.58) | <0.0001 | 5.01 (4.14-6.06) | <0.0001 |
| Chronic respiratory disease | 1.55 (1.38-1.75) | <0.0001 | 0.46 (0.25-0.86) | 0.02 | 1.61 (1.21-2.16) | 0.001 |
| Heart disease | 1.59 (1.41-1.79) | <0.0001 | 0.24 (0.11-0.53) | 0.0004 | 3.55 (2.79-4.50) | <0.0001 |
| Hypertension | 1.53 (1.39-1.69) | <0.0001 | 0.44 (0.28-0.70) | 0.0004 | 3.04 (2.45-3.76) | <0.0001 |
| Diabetes | 1.69 (1.51-1.90) | <0.0001 | 0.72 (0.45-1.17) | 0.19 | 2.62 (2.05-3.36) | <0.0001 |
| Weakened immune system | 2.41 (1.50-3.86) | 0.0003 | - | - | 7.09 (3.52-14.28) | <0.0001 |
| ≥70 years of age | 1.36 (1.24-1.48) | <0.0001 | 0.07 (0.04-0.15) | <0.0001 | 6.53 (5.42-7.88) | <0.0001 |
| ***Shielded*** |  |  |  |  |  |  |
| *Overall* | 1.89 (1.67-2.13) | <0.0001 | 0.23 (0.09-0.56) | 0.001 | 5.62 (4.47-7.07) | <0.0001 |
| Severe respiratory disease | 1.92 (1.66-2.23) | <0.0001 | - | - | 6.16 (4.78-7.93) | <0.0001 |
| Specific cancers | 1.88 (1.41-2.49) | <0.0001 | - | - | 5.00 (3.18-7.87) | <0.0001 |
| Immunosuppressive therapy | 2.15 (1.73-2.69) | <0.0001 | - | - | 5.15 (3.53-7.51) | <0.0001 |
| Solid organ transplant | 2.31 (1.57-3.41) | <0.0001 | - | - | 5.20 (2.71-9.99) | <0.0001 |
| Rare diseases and IEM | 1.49 (1.09-2.03) | 0.01 | - | - | 6.59 (4.39-9.89) | <0.0001 |
| Renal dialysis | 1.61 (1.13-2.30) | 0.009 | - | - | 3.10 (1.61-5.96) | 0.0007 |

*adjusted for sex, deprivation quintile, and other risk categories

**^†^**laboratory-confirmed (test-positive) COVID-19 cases

**^‡^**clinically-confirmed (test-positive or COVID-19 on death certificate) COVID-19 cases

**Supplementary Table 4.** Population attributable fractions for risk categories and risk criteria and COVID-19 outcomes

|  | **Confirmed COVID-19** | | **COVID-19 hospitalisation** | | **COVID-19 ICU admission** | | **COVID-19 Death** | |
| --- | --- | --- | --- | --- | --- | --- | --- | --- |
|  | **%** | **95% CI** | **%** | **95% CI** | **%** | **95% CI** | **%** | **95% CI** |
| ***Moderate*** |  |  |  |  |  |  |  |  |
| *Overall* | 42.06 | 41.49-42.64 | 53.28 | 52.78-53.78 | 22.96 | 17.70-29.09 | 75.30 | 75.09-75.51 |
| Chronic respiratory disease | 3.56 | 3.29-3.83 | 4.84 | 4.58-5.09 | 0.99 | -2.93-5.28 | 2.23 | 2.00-2.50 |
| Heart disease | 4.83 | 4.73-4.94 | 5.97 | 5.86-6.08 | 1.92 | 0.27-3.55 | 6.19 | 6.10-6.28 |
| Hypertension | 5.33 | 5.19-5.45 | 7.06 | 6.93-7.18 | 10.82 | 9.41-12.11 | 4.99 | 4.90-5.09 |
| Diabetes | 10.39 | 10.17-10.58 | 12.96 | 12.77-13.15 | 9.87 | 7.09-12.63 | 12.00 | 11.82-12.18 |
| Weakened immune system | 0.15 | 0.13-0.16 | 0.26 | 0.24-0.28 | - | - | 0.34 | 0.32-0.36 |
| ≥70 years of age | 17.81 | 17.62-18.01 | 22.19 | 21.98-22.43 | - | - | 49.55 | 49.18-49.94 |
| ***High*** |  |  |  |  |  |  |  |  |
| *Overall* | 7.62 | 7.45-7.79 | 12.70 | 12.46-12.90 | 2.69 | 1.44-4.10 | 13.22 | 13.02-13.39 |
| Severe respiratory disease | 3.35 | 3.24-3.46 | 6.08 | 5.97-6.20 | - | - | 7.01 | 6.89-7.11 |
| Specific cancers | 0.88 | 0.84-0.92 | 1.42 | 1.37-1.48 | - | - | 1.31 | 1.27-1.36 |
| Immunosuppressive therapy | 1.41 | 1.36-1.47 | 2.43 | 2.36-2.51 | - | - | 2.05 | 1.99-2.11 |
| Solid organ transplant | 0.39 | 0.37-0.42 | 0.73 | 0.68-0.77 | - | - | 0.57 | 0.54-0.60 |
| Rare diseases and IEM | 0.84 | 0.79-0.88 | 1.17 | 1.11-1.23 | - | - | 1.72 | 1.64-1.81 |
| Renal dialysis | 0.75 | 0.68-0.82 | 0.86 | 0.78-0.94 | - | - | 0.55 | 0.50-0.61 |

 CI confidence interval; IEM inborn errors of metabolism

**Supplementary Figure 1.** Cumulative population attributable fractions (lines) and population prevalence (bars) for individual risk criteria

%s, including prevalence, shown are cumulative. For example there were 10.6% of the population aged 70 or older, and 17.3% aged 70 or older or have hypertension.

Brown – shielded

Pale orange – moderate-risk
